# Structural characterization of borneol dehydrogenase from *Pseudomonas* sp. TCU-HL1

**DOI:** 10.1107/S2053230X20008584

**Published:** 2020-07-01

**Authors:** Aye Aye Khine, Hao-Ping Chen, Kai-Fa Huang, Tzu-Ping Ko

**Affiliations:** a Department of Biochemistry, School of Medicine, Tzu Chi University, Hualien 97004, Taiwan; b Institute of Medical Sciences, School of Medicine, Tzu Chi University, Hualien 97004, Taiwan; c Department of Biochemistry, University of Medicine 1, Yangon, Myanmar; d Integration Center of Traditional Chinese and Modern Medicine, Hualien Tzu Chi Hospital, Hualien 97002, Taiwan; e Institute of Biological Chemistry, Academia Sinica, Taipei 11529, Taiwan

**Keywords:** plant terpenoids, camphor, oxidoreductases, Rossmann fold, NAD, expression medium, protein solubility

## Abstract

During the microbial degradation of borneol, a bicyclic plant monoterpene, it is first converted into camphor by borneol dehydrogenase (BDH) and then enters a known camphor-degradation pathway. Previously, a recombinant *Pseudomonas* BDH was found in inclusion bodies when expressed in *Escherichia coli*. After refolding, it was still unstable and was difficult to concentrate. Here, the protein-expression conditions were improved by changing the medium from lysogeny broth to Terrific Broth, yielding a soluble form of the enzyme with higher activity. The protein was crystallized and its 3D structure was determined by X-ray diffraction. Like other known homologues such as quinuclidinone reductase, the protein forms a tetramer with subunits containing Rossmann folds. Structural comparison revealed major differences in the C-terminal helices and the associated loops. It is likely that these regions contain the determinants for substrate recognition.

## Abstract 2

The structure of a *Pseudomonas* borneol dehydrogenase was determined at 1.84 Å resolution. Major differences from its homologues in the C-terminal helices and the associated loops may suggest determinants for substrate recognition.

## 1. Introduction   

Borneol is one of the terpenoid alcohols that are naturally synthesized in plants and exists in two enantiomeric forms: (+)-borneol and (−)-borneol. (+)-Borneol is also a precursor in the biosynthetic pathway of natural (+)-camphor (Juteau *et al.*, 2002 ▸). Borneol has commonly been used in traditional Chinese medicine for many years. It can relieve pain and serve as an analgesic and anesthetic agent (Hattori, 2000 ▸). Pharmacological effects such as anti-inflammation, neuroprotection and blood–brain barrier penetration have been reported (Liu *et al.*, 2011 ▸; Zhong *et al.*, 2014 ▸; Yin *et al.*, 2017 ▸).

**Figure 1:**
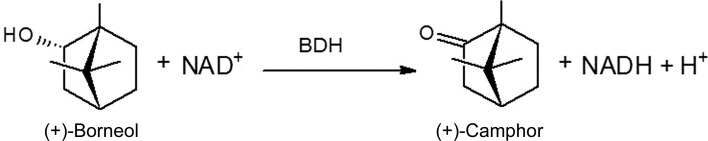
Reaction catalyzed by BDH.

The degradation of borneol in microorganisms has previously been reported by our group (Tsang *et al.*, 2016 ▸). Borneol dehydrogenase (BDH; accession No. WP_069084981) converts borneol into camphor in *Pseudomonas* sp. TCU-HL1 (Fig. 1 ▸). The camphor is then metabolized by this microorganism through a known camphor-degradation pathway (Ougham *et al.*, 1983 ▸; Taylor & Trudgill, 1986 ▸; Leisch *et al.*, 2012 ▸; Iwaki *et al.*, 2013 ▸). The gene encoding this enzyme, *bdh*, with locus tag TCL1_4180, was found in the camphor-degradation gene cluster. Recombinant BDH was produced in the form of inclusion bodies in *Escherichia coli* BL21(DE3) cells. However, even after refolding, it was unstable and very easily precipitated. Consequently, various protein-induction methods have been tested in order to improve the solubility of this recombinant enzyme.

The BDH protein is homologous in primary sequence to a number of other oxidoreductases for which three-dimensional structures are available in the Protein Data Bank. The most similar is the 3-quinuclidinone reductase from *Agrobacterium tumefaciens* (AtQR; 36.4% identity; PDB entries 3ak4 and 3wds; Hou *et al.*, 2014 ▸). The enzyme assembles into a tetramer of four identical subunits; each subunit contains a central parallel β-sheet with flanking α-helices. Here, to provide a structural basis for future design and engineering to modify the substrate specificity of BDH, we crystallized the protein and determined its 3D structure.

## 2. Materials and methods   

### 2.1. Macromolecule production   

The expression vector pbdhX, derived from pET-30 (Tsang *et al.*, 2016 ▸), was used to transform *E. coli* BL21(DE3) cells for recombinant BDH production. The bacterial growth and induction conditions were the same as reported previously, except that Terrific Broth (TB) was used instead of lysogeny broth (LB). All purification steps were carried out at 4°C. About 10 g of cells (wet weight) were resuspended in 50 ml 10 m*M* sodium phosphate buffer pH 7.0, 1 m*M* phenylmethylsulfonyl fluoride (PMSF), 1 m*M* dithiothreitol (DTT) and were ruptured by sonication. The crude extracts were clarified by centrifugation at 25 000*g* for 15 min. The supernatant was brought to 20% ammonium sulfate saturation, clarified by centrifugation and loaded onto a Phenyl Sepharose High Performance hydrophobic interaction column (2.6 × 70 cm) pre-equilibrated with 400 ml buffer *A* (1 *M* ammonium sulfate, 10 m*M* sodium phosphate pH 7). The proteins were eluted with buffer *A* from 0 to 50 ml, with 100% buffer *A* to 100% buffer *B* (10 m*M* sodium phosphate pH 7) from 50 to 1050 ml and with buffer *B* from 1050 to 1250 ml. The BDH activity was determined using the procedure reported previously (Tsang *et al.*, 2016 ▸), and fractions containing BDH activity were pooled. After overnight dialysis against buffer *B* with 1 m*M* DTT, the protein solution was loaded onto a Q Sepharose Fast Flow anion-exchange column (2.6 × 50 cm) and eluted using the method described above, in which buffer *A* was replaced by buffer *B* and the second buffer was buffer *B* with 0.5 *M* KCl. Macromolecule-production information is summarized in Table 1 ▸.

### 2.2. Crystallization   

For crystallization experiments, the purified BDH was dialyzed and concentrated to ∼10 mg ml^−1^ in a new buffer consisting of 50 m*M* Tris–HCl pH 8.0, 5% glycerol. An initial crystallization screening of ∼800 conditions was performed at the Protein Crystallization Facility, Institute of Biological Chemistry, Academia Sinica, Taipei, Taiwan. Single crystals were found in PEG-containing drops. After optimization, diffraction-quality crystals were obtained using a reservoir solution consisting of 30%(*w*/*v*) PEG 8000, 0.1 *M* sodium acetate pH 4.5, 0.2 *M* lithium sulfate. The crystals were grown at 20°C by the sitting-drop vapor-diffusion method. Crystallization information is summarized in Table 2 ▸.

### 2.3. Data collection and processing   

The crystals were first analyzed using the in-house X-ray facility at the Institute of Biological Chemistry, Academia Sinica. They belonged to the orthorhombic space group *F*222, with one BDH molecule in the asymmetric unit. High-resolution X-ray diffraction data collection was carried out on the Taiwan Light Source (TLS) beamline BL15A1 at the National Synchrotron Radiation Research Center (NSRRC), Hsinchu, Taiwan. Prior to flash-cooling for data collection, the BDH crystals were soaked briefly in reservoir solution containing 10–15%(*v*/*v*) glycerol as a cryoprotectant. In an attempt to obtain complex crystals, 5 m*M* NAD was added to the reservoir solution. The diffraction intensities were integrated and scaled using *HKL*-2000 (Otwinowski & Minor, 1997 ▸). Data-collection and processing statistics are summarized in Table 3 ▸.

### 2.4. Structure solution and refinement   

The crystal structure was solved using *MOLREP* (Vagin & Teplyakov, 2010 ▸) from *CCP*4 (Winn *et al.*, 2011 ▸) with PDB entry 3ak4 as a search model and was refined to 1.84 Å resolution using *Phenix* (Liebschner *et al.*, 2019 ▸) and *Coot* (Emsley *et al.*, 2010 ▸). Use of translation–libration–screw (TLS) parameters did not improve the *R* values, and therefore all atoms were treated with isotropic temperature factors. *MolProbity* (Chen *et al.*, 2010 ▸) was used for Ramachandran analysis. In addition to the NAD from PDB entry 3ak4, a model of camphor (CAM) was generated and both were placed into the active site of BDH using *Phenix* and *Coot*. Among the top 100 BDH-like structures in the PDB found by the *DALI* server (Holm, 2019 ▸), only a few contained bound substrates. These served as a guide for positioning the CAM model, which was subsequently improved by repeated manual adjustment and regularization according to the known structures and stereochemical restraints. In particular, the carbonyl group of CAM was oriented for the correct handedness of borneol upon hydride transfer. All figures showing 3D protein structures were produced using *PyMOL* (https://pymol.org/). Refinement statistics are summarized in Table 4 ▸.

## 3. Results and discussion   

### 3.1. Expression of soluble BDH   

Most of the recombinant BDH was produced in a soluble form in crude extracts of *E. coli* using Terrific Broth as the growth medium. This was in contrast to the previous results using LB medium, in which the recombinant BDH was found in inclusion bodies and required refolding to solubilize it (Tsang *et al.*, 2016 ▸). The specific activity of the soluble form of BDH turned out to be about 40-fold higher than that of the refolded enzyme. A homogeneous preparation of the enzyme was obtained after purification steps using hydrophobic and anion-exchange columns (Supplementary Fig. S1). The purified BDH was very stable and did not precipitate upon subsequent dialysis and concentration.

### 3.2. Crystal structure of BDH   

**Figure 2:**
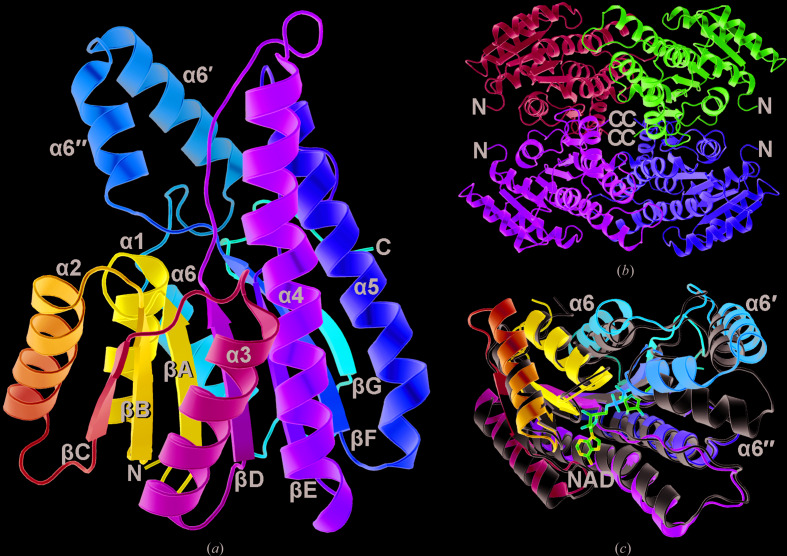
Overall structure of BDH. (*a*) The BDH monomer is shown as a ribbon diagram colored blue–cyan–green–yellow–red from the N-terminus to the C-­terminus. The α-helices and β-strands are denoted by numbers and letters. (*b*) The four monomers in a BDH tetramer are colored differently, with the N- and C-termini indicated. (*c*) The structure of BDH, presented as in (*a*), is compared with that of AtQR (PDB entry 3ak4), which is colored gray. Most of the Rossmann-fold part superimposes well, whereas helices α6′′, α6′ and α6 deviate significantly. The bound NAD in AtQR is shown as a stick model.

The refined model of the orthorhombic BDH crystal contains an unbroken polypeptide chain from the N-terminal Lys2 to the C-terminal Arg260. As shown in Fig. 2 ▸(*a*), the protein features a seven-stranded antiparallel β-sheet flanked by three α-helices on each side, making up a Rossmann-fold structure. The longer helices α4 and α5 on one side protrude over the C-terminal ridge of the β-sheet, where the cofactor NAD is supposed to bind. On the other side, two additional helices α6′′ and α6′ are inserted between strand βF and helix α6. The space surrounded by these four α-helices should participate in substrate binding and catalysis. However, soaking experiments failed to show electron density for NAD.

Although the *F*222 crystal only contains one BDH protomer in the asymmetric unit, a similar tetramer to those of AtQR and other homologous oxidoreductases can be generated by crystallographic symmetry operations, as shown in Fig. 2 ▸(*b*). The protein–protein interface buries 3520 Å^2^ (28%) of the total 12 520 Å^2^ surface area of each protomer. The tetramers of BDH and AtQR (PDB entry 3ak4) superimpose well, with an overall root-mean-square deviation (r.m.s.d.) of 1.69 Å for 964 pairs of matched C^α^ atoms (Supplementary Fig. S2). If individual protomers are compared, the r.m.s.d. ranges from 1.52 to 1.58 Å for 239–242 C^α^ pairs. The regions that differ the most are located in helices α6′′, α6′ and α6 and the associated loops, as shown in Fig. 2 ▸(*c*).

### 3.3. Substrate-binding mode   

**Figure 3:**
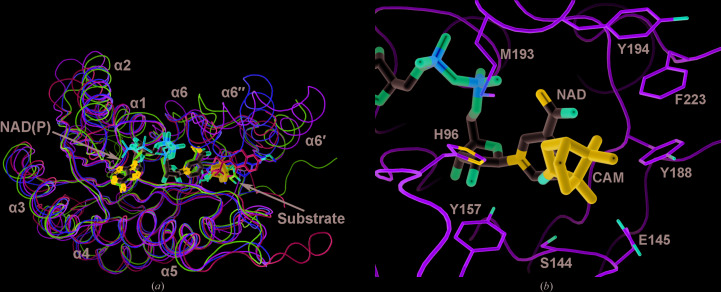
Substrate-binding region. (*a*) The monomers of BDH (with acetate; green) amd PDB entries 1e6w (rat HADH II/ABAD + estradiol; cyan), 3o03 (*Streptococcus* Ga5DH + gluconate; magenta) and 6ihh (*Ralstonia* cyclodiketone reductase + ethyl secodione; yellow) are superimposed and shown as wire models, along with the active-site-bound ligands, which are shown as thin sticks. The NAD from AtQR (gray) and the modeled camphor (blue) are shown as thick sticks. The BDH helices are labeled. (*b*) The cofactor- and substrate-binding region of BDH is shown in a close-up view. Amino-acid side chains surrounding the substrate are shown as thin sticks.

The nicotinamide moiety of NAD from AtQR lies at the bottom of a cavity in BDH, surrounded by loop βD–α4, loop βE–α5, loop βF–α6′′ and helix α6′′ (Supplementary Fig. S3). While the NAD-binding site is largely conserved, the surrounding regions are variable, which should account for the binding to the preferred substrate. In this regard, three substrate-containing structures, PDB entries 1e6w (Powell *et ;al.*, 2000 ▸), 3o03 (Zhang *et al.*, 2009 ▸) and 6ihh (Chen *et al.*, 2019 ▸), were compared and a BDH substrate was modeled into the active site (Fig. 3 ▸). The plausible binding mode suggests hydrogen bonding between the His96 and Tyr157 side chains and the borneol hydroxy group, as well as nonpolar contacts of the Phe233 side chain with the dimethyl group. However, the precise enzyme–substrate interactions have yet to be revealed by further investigations.

## Supplementary Material

PDB reference: borneol dehydrogenase, 6m5n

Supplementary Figures. DOI: 10.1107/S2053230X20008584/no5178sup1.pdf

## Figures and Tables

**Table 1:** Macromolecule-production information

Source organism	*Pseudomonas* sp. TCU-HL1
DNA source	TCU-HL1 genomic DNA
Forward primer†	5′-ATTTAGACATATGAAACTGCTAGAAGGTAAAAGAATCATC-3′
Reverse primer‡	5′-TAAGCGGATCCTAGCGTACAGCGTACAGCGATCAGGCCAT-3′
Cloning vector	pET-30a
Expression vector	pET-30a
Expression host	*E. coli* BL21(DE3)
Complete amino-acid sequence of the construct produced	MKLLEGKRIIVTGGAQGIGASVVRAYIAAGATVASMDMNDTLGQQVVSEAGKANPGCKSRYYHCNIADRPEVEKAFATAAEDMGGLDVMVNVAGVHRHSPPDAIAEELYDMLFRVNVLGTINTNAVAYRLMKGQGIGNIINFGSESGLTGEINNALYSATKAAVHTWTRNVARQWGPDGIRINAVLPYMVTPMYVDFRNALSSEDLAAHDAATKTDIPLGGKFGDADKDLAPVMVFLASDASHFMTGQMFPVDGGLIAVR

† The NdeI site is underlined.

‡ The BamHI site is underlined.

**Table 2:** Crystallization

Method	Sitting-drop vapor diffusion
Plate type	Intelli-Plate (Art Robbins Instruments)
Temperature (K)	293
Protein concentration (mg ml^−1^)	10
Buffer composition of protein solution	50 m*M* Tris–HCl pH 8.0, 5% glycerol
Composition of reservoir solution	30%(*w*/*v*) PEG 8000, 0.1 *M* sodium acetate pH 4.5, 0.2 *M* lithium sulfate
Volume and ratio of drop	5 µl protein solution and 5 µl reservoir solution (1:1)
Volume of reservoir (µl)	50

**Table 3:** Data collection and processing Values in parentheses are for the outer shell.

Diffraction source	NSRRC beamline BL15A1
Wavelength (Å)	1.0000
Temperature (K)	100
Detector	Rayonix MX300HE CCD
Space group	*F*222
*a*, *b*, *c* (Å)	71.63, 96.51, 142.56
α, β, γ (°)	90, 90, 90
Resolution range (Å)	30.000–1.840 (1.91–1.84)
Total No. of reflections	107237
No. of unique reflections	21642 (2120)
Completeness (%)	100.0 (100.0)
Multiplicity	5.000 (5.00)
Average *I*/σ(*I*)	11.54 (2.03)
Average CC_1/2_	0.918 (0.679)
*R*_merge_	0.137 (0.842)
*R*_p.i.m._	0.067 (0.413)
Overall *B* factor from Wilson plot (Å^2^)	20.51

**Table 4:** Structure refinement Values in parentheses are for the outer shell.

Resolution range (Å)	25.55–1.84 (1.90–1.84)
Completeness (%)	99.4 (95.6)
No. of reflections, working set	20451 (1930)
No. of reflections, test set	1076 (101)
Final *R*_cryst_	0.157 (0.232)
Final *R*_free_	0.182 (0.274)
No. of non-H atoms	
Protein	1927
Ligand	17
Water	285
R.m.s. deviations	
Bond lengths (Å)	0.005
Angles (°)	0.957
Average *B* factors (Å^2^)	
Protein	19.8
Ligand	44.4
Water	32.3
Ramachandran plot	
Favored regions (%)	97.3
Additionally allowed (%)	2.3
Clashscore	2.07
*MolProbity* score	1.19
