# Supplementary material for: Structural characterization of borneol dehydrogenase from Pseudomonas sp. TCU-HL1
Source: Acta Crystallogr F Struct Biol Commun. 2020 Jul 1;76(Pt 7):309–13. doi: 10.1107/S2053230X20008584 (PMC7336358; doi:10.1107/S2053230X20008584)
Supplement: Supplementary file 1 [file f-76-00309-sup1.pdf]

Acta Cryst F

STRUCTURAL BIOLOGY COMMUNICATIONS

Volume 76 (2020)

Supporting information for article:

Structural characterization of borneol dehydrogenase from *Pseudomonas* sp. TCU-HL1

Aye Aye Khine, Hao-Ping Chen, Kai-Fa Huang and Tzu-Ping Ko

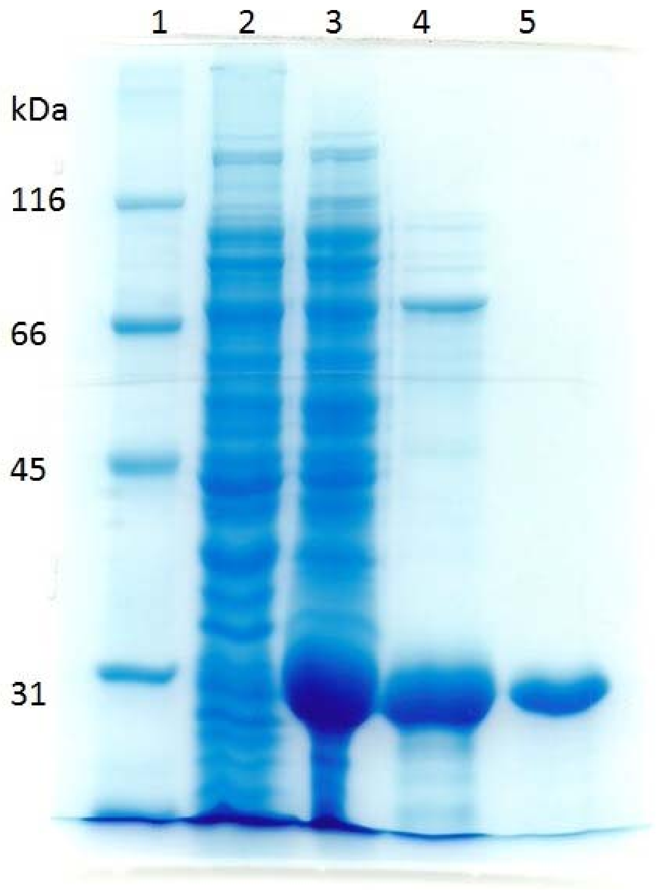

Figure S1 SDS-PAGE showing the purification steps of recombinant BDH (in 10.5% gel). Lane 1: marker; Lane 2: crude extract before IPTG induction; Lane 3: crude extract after IPTG induction; Lane 4: fraction after hydrophobic chromatography; Lane 5: fraction after ion-exchange chromatography.

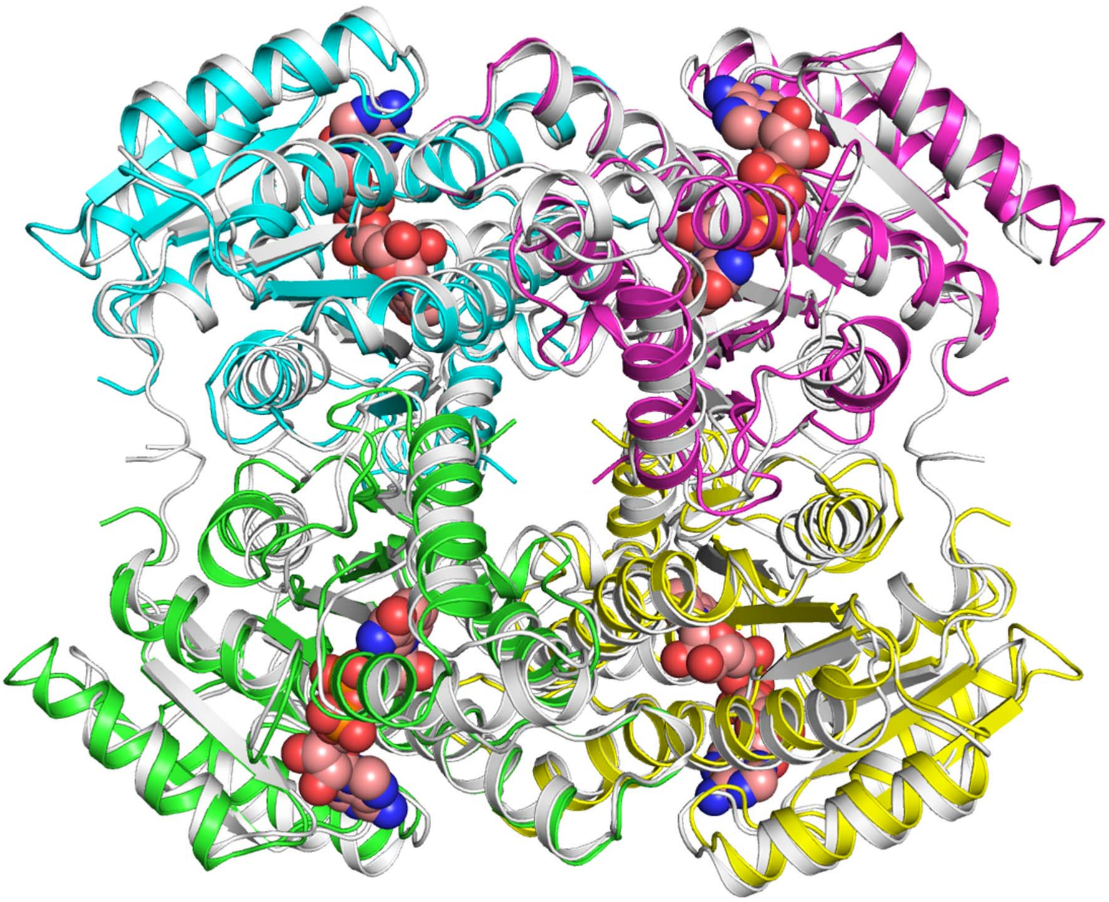

Figure S2 Structural comparison of tetramers. BDH is colored green, cyan, magenta and yellow, whereas AtQR is in gray. The bound NAD molecules in AtQR are shown as sphere models with pink carbon atoms.

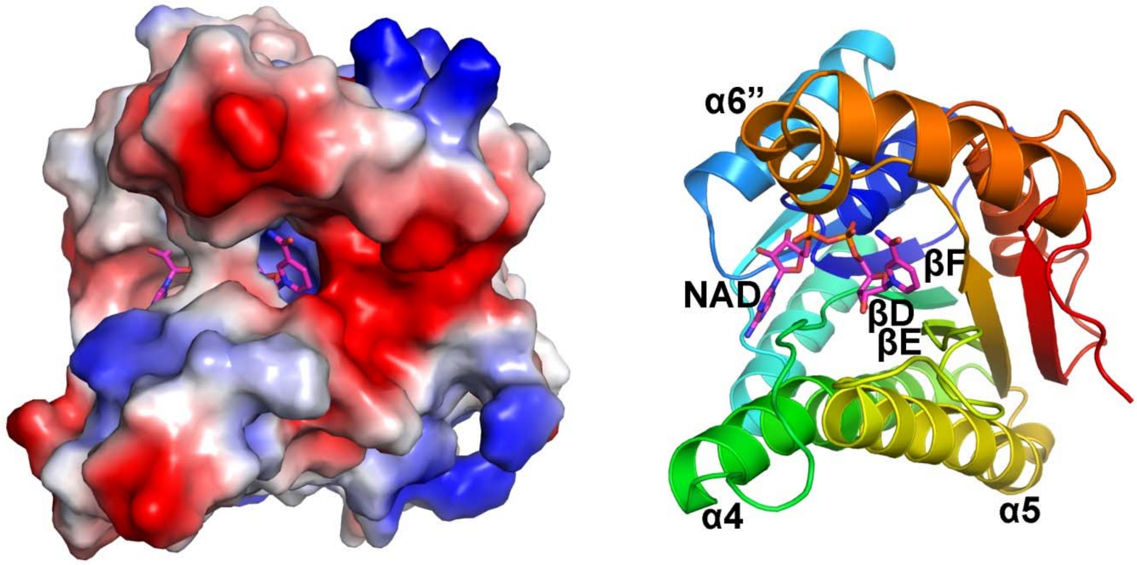

Figure S3 Active site of BDH. On the left is a surface electrostatic potential representation of the BDH monomer, in which the NAD from AtQR is also shown, as a magenta stick model. Positively and negatively charged surface areas are colored blue and red. Neutral areas are white. On the right is a ribbon diagram of the same BDH and NAD model. Related secondary structural elements to substrate binding are labeled.
